# The complex treatment including rituximab in the Management of Catastrophic Antiphospholid Syndrome with renal involvement

**DOI:** 10.1186/s12882-018-0928-z

**Published:** 2018-06-08

**Authors:** Aleksandra Rymarz, Stanisław Niemczyk

**Affiliations:** 1 Department of Internal Diseases, Nephrology and Dialysis, Military Institute of Medicine, Szaserów 128, 04-141 Warsaw, Poland; 2 Department of Internal Diseases, Nephrology and Dialysis, Military Institute of Medicine, Szaserów 128, 04-141 Warsaw, Poland

## Abstract

**Background:**

Catastrophic antiphospholipid syndrome (CAPS) is a rare, life-threatening form of antiphospholipid syndrome (APS) involving many organs and leading to their insufficiency. The pathogenesis of CAPS is associated with the presence of antiphospholipid antibodies (aPL). Typical therapy includes anticoagulation, glucocorticoids, therapeutic plasma exchanges and/or intravenous immunoglobulin. Despite this aggressive treatment, the mortality rate of 37% is still high. Novel therapeutic agents are required. Rituximab (RTX) is the most studied drug in APS also used in CAPS. Because of the rarity of CAPS occurrence it is impossible to plan a controlled, randomized study exploring its efficacy in CAPS. Therefore, case reports of its usage can be a source of our knowledge in this matter.

**Case presentation:**

A 35-year-old woman who displayed dyspnoea and peripheral edema was admitted to the Nephrology Clinic because of rapidly progressive renal insufficiency. Her history included autoimmune hemolytic anemia anemia, two miscarriages and the diagnosis of APS with the treatment of heparin and acetylosalicylic acid during her next pregnancy. In spite of this treatment, she gave birth to a dead fetus in 22 Hbd. She then developed CAPS with involvement of the kidneys, brain, skin, peripheral veins and central retinal artery. Lupus anticoagulant and β_2−_glicoprotein-I antibodies were positive. Immediately upon admission to the nephrology clinic, she received anticoagulation and corticosteroids along with therapeutic plasma exchanges. As a supportive treatment hemodialysis sessions were necessary. Under this treatment the amelioration of the general state was observed but renal failure persisted, therefore intravenous immunoglobulin was added. Afterwards, the kidney function recovered and the renal replacement therapy could be stopped. After this therapy, aPL became negative. Four weeks later lupus anticoagulant began to increase. Taking into account the risk factors of the relapse and the life-threatening course of the disease, rituximab was introduced. After administration of 2 g of RTX in three separate doses, we observed no new thrombotic events, the further amelioration of renal function and the negative profile of aPL.

**Conclusions:**

CAPS is a life-threatening condition and a prompt, complex treatment is necessary. Rituximab together with conventional therapy can be an additional option in case of the risk of relapse.

## Background

Catastrophic antiphospholipid syndrome (CAPS) is a rare, life-threatening form of antiphospholipid syndrome (APS) affecting around 1% of all patients with APS. Its pathogenesis is associated with the presence of antiphospholipid antibodies (aPL) represented by anti β_2−_glicoprotein-I antibodies (aβ_2_GPI), anticardiolipin antibodies (aCL) and lupus anticoagulant (LA). These autoantibodies activate endothelial cells, platelets, immune cells and the complement system. This action induces intravascular thrombosis enhanced by the inhibition of anticoagulants and the fibrinolysis process [1]. The activation of immune cells is associated with cytokine storm and the development of systemic inflammatory response syndrome (SIRS) [2]. In recent times, the role of high levels of ferritin as a pro-inflammatory factor has been discussed. CAPS is postulated as one of the clinical conditions included in the hyperferritinemic syndrome [3]. Authors have suggested that an elevated ferritin level is not only a reflection of inflammation as an acute phase response but also an immunomodulatory molecule which contributes to the pathogenic mechanism of CAPS.

The clinical manifestation of CAPS is associated with arterial and/or venous thrombosis in vessels supplying at least three organs and resulted in their ischemia and failure. The diagnosis of CAPS can be set if the symptoms develop within 1 week [4]. The criteria was originally proposed by Asherson in 2002 and finally validated in 2005 [5, 6]. The most commonly affected organs include the kidneys (73%), lungs (60%), central nervous system (56%), heart (50%), skin (47%), liver (39%), peripheral vessels (37%), gastrointestinal tract (24%) [7]. The presence of aPL is not sufficient to activate coagulation storm. An additional factor called ‘second hit’ must also appear. In the recent report the ‘second hit’ was identified in 65% of patients with CAPS [7]. Among precipitating factors, infections, surgery, cessation of anticoagulation, drugs, obstetric complications, neoplasms should be enumerated [8]. APS can be a primary disease, but in 40% of cases it is concomitant to lupus erythematosus. Although kidneys at the presentation are affected only in 18% of cases, eventually 71% of patients experience kidney involvement. The manifestation of kidney disease is hypertension, proteinuria, hematuria, renal failure expressed by elevation of serum creatinine level and less frequently renal vein thrombosis and renal artery thrombosis. The essence of CAPS is microcirculation involvement and a typical histological demonstration is acute thrombotic microangiopathy. Similar changes also occur in haemolytic-uremic syndrome, thrombotic thrombocytopenic purpura, disseminated intravascular coagulation and heparin-induced thrombocytopenia, and these disorders should be taken into account in the differential diagnosis.

Considering that CAPS is a life-threatening event, therapeutic strategies should be applied quickly. Complex treatment directed against the thrombotic event and inflammation is also required. The anticoagulation (AC) with heparin, glucocorticoids (GCS), therapeutic plasma exchanges (TPE), immunoglobulin (IVIG) along with supportive treatment like hemodialysis or mechanical ventilation support are possible therapeutic options. According to the newest CAPS registry report the most frequently used combination is AC plus GCS (19%), followed by AC plus GCS plus TPE or IVIG (18%) [7]. The enhancement of therapy by TPE and/or IVIG reduces the mortality rate. Among 342 patients with CAPS, 160 who received triple therapy including TPE or/and IVIG had better survival than those on the double treatment (AC and GCS) [9]. In the case of CAPS secondary to systemic lupus erythematosus (SLE), cyclophosphamide administration is strongly recommended [8]. Despite advanced therapeutic strategies, the mortality rate is still at a high level of 37% [7]. Novel medications like rituximab or eculizumab are used in refractory and relapsing cases. Rituximab (RTX) humanized anti-CD20 monoclonal antibody primary used in B-cell malignancies is currently widely applied in many autoimmune disorders. According to CAPS registry, out of 20 patients treated with RTX, 15 recovered [10]. Eculizumab is a humanized monoclonal antibody directed against complement C5 convertase which prevents its activation. It is applied in atypical hemolytic-uremic syndrome and paroxysmal nocturnal hemoglobinuria. Animal models of APS thrombosis showed that complement activation plays a significant role in this phenomenon [11]. Knowledge about the utilization of eculizumab in humans is based on the case studies where this drug has been used in refractory cases [12].

## Case presentation

**Fig. 1:**
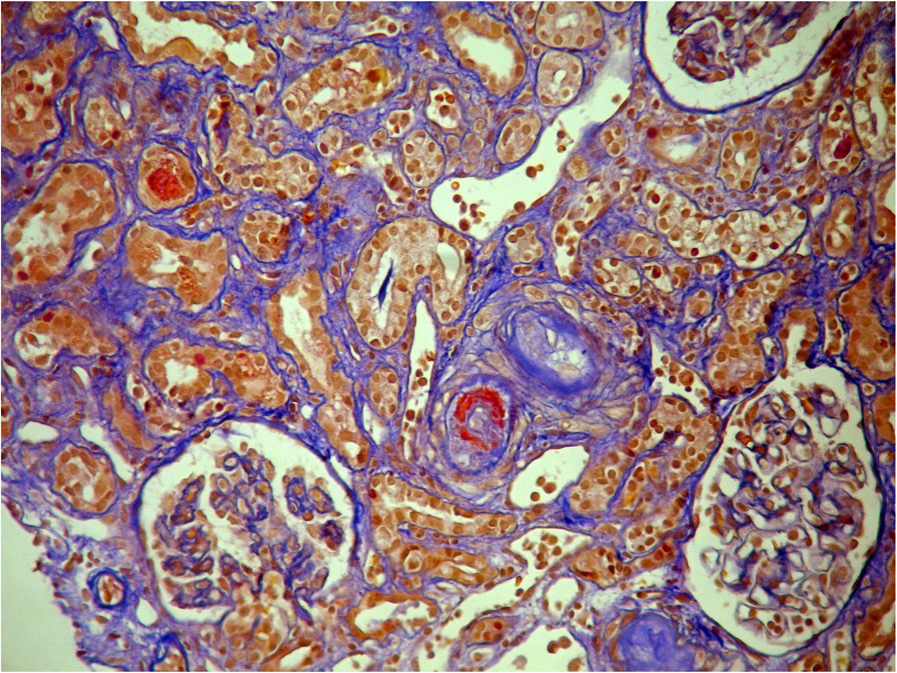
Renal biopsy presenting fibrinoid necrosis of the vessel wall associated with thrombotic microangiopathy

A 35-year-old woman who displayed dyspnoea and peripheral edema was admitted to the Nephrology Clinic because of rapidly progressive renal insufficiency. Her history included autoimmunohemolytic anemia, two miscarriages and the diagnosis of APS with the treatment of heparin and acetylosalicylic acid (ASA) during her next pregnancy. The diagnosis of APS after the first miscarriage was supported by positive serological tests such as lupus anticoagulant, anti β2glicoprotein-1 antibodies class IgG and anticardiolipn antibodies class IgM and IgG. Despite this treatment, in 22 hbd ultrasound no heart function of the morphological normal fetus was revealed. After the dead fetus was delivered, there appeared edema of the legs, a reduction in the amount of urine and blurred vision. Ultrasounds with power Doppler of the lower limbs revealed deep vein thrombosis. The ophthalmic examination revealed the right central retinal artery thrombosis. The computer tomography (CT) scan of the head presented an ischemic stroke in the right temporal lobe. The CT scan of the chest only revealed a high amount of fluid in both pleural cavities without any abnormalities in the pulmonary veins or arteries. The clinical symptoms were accompanied by the following laboratory abnormalities: anaemia (Hgb - 7 g/dl), thrombocytopenia 134 G/l, leucocytosis (WBC) 14.36 G/l, elongate activated partial thromboplastin time (APTT) 69.7 s (reference range 23–35 s), elevated D-dimer level (4.33 μg/ml, reference range 0–0.5), elevated serum creatinine (4.8 mg/dl) and urea (79 mg/dl) levels. Fibrinogen level (247 mg/dl) and antithrombin activity (101%) were within the reference range. All of the described symptoms appeared within 1 week. Because of the deterioration of her general state - aggravating dyspnoea and peripheral oedema with concomitant rapidly progressive renal failure - the patient was moved to the Nephrology Clinic. Respiratory failure with mouth cyanosis, a vast amount of fluid in both pleural cavities, massive peripheral oedema and livedo reticularis on the skin were observed upon the patient’s admission. Laboratory findings included elevated creatinine (5,5 mg/dl) and urea level 92 mg/dl, Hgb 8.7 g/dl, PLT 143 G/l, WBC 13.6 G/l, daily proteinuria 0.7 g, erytrocyturia, hypoproteinemia (5 g/dl), hypoalbuminemia (2.5 g/dl), an elevated level of lactate dehydrogenase (548 U/L, reference range 135–223), elevated C-reactive protein level (4.7 mg/dl, reference range < 0.8), elevated ferritin level 308 ng/ml (reference range 30–200). Levels of complement components were C3–77 mg/dl (reference range 90–180), C4–10 mg/dl (reference range 10–40). Antibodies against β_2_ glicoprotein I were elevated (14 U/ml), the significant content of lupus anticoagulant was also revealed. Antinuclear antibodies (ANA), anti-double strength DNA antibodies, c-ANCA p-ANCA, anticardiolin antibodies (aCL) were negative. ADAMTS 13 and its inhibitor were normal. Abdominal ultrasound revealed the enlargement of both kidneys (left kidney 139 mm, right kidney 124 mm) with interstitial edema and left renal vein thrombosis. An X-ray of the chest demonstrated a high amount of fluid in both pleural cavities. Histopathological examination showed thrombotic microangiopathy (TMA) secondary to APS [Fig. 1].

Clinical signs, organ manifestation and laboratory findings are summarized in Table 1.

**Table 1:** Clinical evolution of CAPS in the described patient

Organ manifestation	Clinical signs	Laboratory abnormalities typical for CAPS	Laboratory tests which were normal
Kidneys			
renal failure	Peripheral edema	Thrombocytopenia	Fibrinogen
renal vein thrombosis	Fluid in both pleural cavities	Elongate APTT	ANA
hematuria		Elevated D-dimer	Anti-dsDNA
proteinuria		Reduced complement components C3 and C4	ADAMTS 13 and its inhibitor
Brain			
stroke		Positive lupus anticoagulant	
Skin			
livedo reticularis	livedo reticularis	Antiβ2glicoprotein-I antibodies	
Peripheral vessel			
peripheral venous thrombosis	Edema of the legs	Thrombotic microangiopathy in renal biopsy	
Eye			
central retinal artery thrombosis	Blurred vision		

*APTT* activated partial thromboplastin time, *ANA* antinuclear antibodies, *anti-dsDNA* anti-double strength DNA antibodies, *CAPS* catastrophic antiphospholipid syndrome

Because of thrombosis of vasculature in more than three organs within a short period of time, the diagnosis of catastrophic antiphospholipid syndrome was made. Anticoagulation treatment with infusion of high molecular weight heparin was introduced. The dosage was modified according to APPT with a target level of 70–85 s. Glucocorticoids: methyprednisolon 1 g i.v. for three consecutive days, followed by prednisone 1 mg/kg/day (60 mg/day) were also used. Therapeutic plasma exchanges were added to remove antiphospholipid antibodies. Because of severe renal insufficiency with anuria, fluid overload with respiratory failure, daily hemodialysis treatment was introduced. Systemic antibiotic therapy with piperacyllin/tazobactam was also added. Under this treatment, an evident improvement was observed. Dyspnoea and the respiratory failure gradually disappeared, the haemoglobin level increased, the platelet amount normalized but oliguria still remained and regular hemodialysis sessions were necessary. High molecular weight heparin was replaced by the low molecular weight heparin (enoxaparin 60 mg). TPE were halted after five sessions. Because of the lack of amelioration of the kidney function, it was decided to enhance the treatment and intravenous immunoglobulin was introduced. The patient received five doses of IgG 0.4 g/kg/day. The CAPS treatment of this patient was summarized in Table 2.

**Table 2:** Treatment of CAPS used in the described patient

First line treatment	Anticoagulation	High molecular weight heparin (HMWH)
		LMWH
		Oral anticoagulant (warfarin, INR > 3)
	Glucocorticoids	Methylprednisolone 1 g i.v. for three consecutive days
		Oral prednisone, 1 mg/kg/day (60 mg/day)
		Oral prednisolone tapered to 20 mg within 6 weeks
	TPE	5 sessions with the frequency every other day
		1,5 of total plasma volume per session
		Replacement fluid: 4% albumin solution and FFP
	Immunoglobulin	5 doses of IgG 0,4 g/kg/day
Second line treatment	Rituximab	2 g in three separate doses (600–800-600 mg) in two-month intervals

*FFP* fresh frozen plasma, *LMWH* low molecular weight heparin

In the meantime, the temporary catheter was removed and a permanent catheter was introduced. The intervention was complicated by a haemorrhage in the place of the insertion. A few days later, fever, chills and elevated inflammatory markers disclosed a catheter induced infection. Ceftriaxon and linezolid were introduced with a positive response. The permanent catheter was replaced by another temporary one.

Three weeks after immunoglobulin treatment, a gradual increase in the amount of urine and a decrease in serum creatinine and urea levels were observed. Renal replacement therapy could be discontinued after 5 weeks of treatment. The patient was discharged from the hospital with the creatinine level 3.2 mg/dl, urea level 157 mg/dl, concomitant erytrocyturia 250 cells/μl, daily proteinuria 0.5 g. All antiphospholipid antibodies were negative. The treatment at the discharge from the hospital was: oral anticoagulant (warfarin), prednisolon (20 mg/day), ASA and antihypertensive drugs.

After 4 weeks (3 months after the first symptoms of CAPS occurred) the control laboratory tests revealed medium positive lupus anticoagulant, creatinine level was 2.5 mg/dl, urea level 142 mg/dl. Fearing a CAPS relapse, after the acceptance of the multidisciplinary committee, rituximab therapy was introduced. Before administering the drug, an infection screening was performed. HIV, hepatitis B and C, and tuberculosis markers were negative. Blood and urine cultures were also negative. The patient received 2 g of RTX in three separate doses (600–800-600 mg) in two-month intervals. During the therapy, the infection prophylaxis with trimetoprim/sulfametoxazol and acyclovir was used. Six months after the last dose of RTX, the serum creatinine level was 1.6 mg/dl, urea level 40 mg/dl, erytrocyturia 250 cells/μl, daily proteinuria 0.2 g. All antiphospholipid antibodies were negative. One year after the last dose of RTX, LA increased from negative to slightly positive but the renal function remained good with serum creatinine level 1.4 mg/dl and no new thrombotic events observed. Prednisone was stopped 2 years after the CAPS presentation and 15 months after the last dose of RTX.

## Discussion

CAPS is a rare yet serious autoimmune disorder. The most latest CAPS registry report, noted 500 patients of which 69% were female. There are some differences in epidemiology between primary and secondary CAPS. Patients with primary CAPS are older (mean age 39.8 vs. 32.8 years) and have a lower mortality rate (33 vs. 48%) than with secondary CAPS. Our female patient with primary CAPS was 35 years old. However, the most frequent precipitating factors are infections - in the presented case obstetric complication induced CAPS. Pregnancy related CAPS represent 8% of all CAPS cases [7]. Pregnancy loss is linked to the activation of complement cascade which plays a significant role in CAPS pathogenesis [13].

The most frequently affected organs are the kidneys, lungs, brain, heart, skin, liver and peripheral vessels. The diagnosis of CAPS requires the involvement of at least three organs. In the presented case, the kidneys, brain, skin, peripheral veins and central retinal artery were engaged. The clinical manifestation of renal involvement includes renal failure, proteinuria, arterial hypertension, hematuria and less often thrombosis of the renal vein or artery. Renal failure is the most common form of renal involvement resulting from thrombotic microangiopathy in glomeruli. This complication often requires a supplementary treatment such as hemodialysis sessions, as in the case of our patient who suffered from severe renal failure, arterial hypertension, proteinuria, hematuria and renal vein thrombosis.

CAPS is a life-threatening condition which requires complex treatment directed at thrombotic events and the inflammatory state. Anticoagulation with heparin not only inhibits clot formation and promotes fibrinolysis but also prevents aPL from binding to their target on the cell surface and restraining complement activation [14]. Initially non-fractionated heparin and intravenous administration is preferred. The advantages of this kind of treatment are associated with the option of quickly reversing its action in case of bleeding or the necessity of invasive procedures. In long-term therapy, oral anticoagulation is recommended. In order to inhibit inflammation associated with CAPS, glucocorticoids (GCS) are used. GCS inhibit the nuclear factor (NF) - κß, a SIRS mediator through which anti-β_2_GPI antibodies induce coagulation and/or inflammation [15]. There are no evidence based recommendations for the optimal dosage nor the duration of GCS treatment. Traditionally patients receive 1000 mg of methylprednisolone for 3 to 5 days followed by oral prednisone. However, dual therapy with AC and GCS is the most commonly used option, where the recovery rate is 63.8%. The enhancement of treatment with therapeutic plasma exchanges ameliorates the recovery rate to 77.8% [8]. Contrary to immunosuppressors whose action is delayed after their usage, plasma exchanges remove promptly aPL antibodies and proinflammatory mediators such as Il-1, Il-6, Il-18, TNF-α, ferritin. However, these procedures also remove plasma immunoglobulins, sometimes causing their deep deficiency. Decreased plasma immunoglobulins levels are associated with an increased risk of infection and its supplementation can be indicated. Intravenous immunoglobulins (IVIG) are also used in the treatment of CAPS. They have a direct effect through the Fc receptor which enables the autoantibodies blockage and an increase in its clearance. An indirect effect is associated with immunomodulatory action through suppressing cytokines, inhibiting CD8 and inhibiting complement activation. Typical dosage is 0.4 g/kg/day for 5 days administered after the last plasma exchange session. Most analysis - concentrated on the result of CAPS treatment - groups the IVIG together with TPE proving that the triple (AC + GS + TPE or IVIG) or quadruple (AC + GS + TPE + IVIG) therapy is more effective than the double one. There is no analysis comparing the triple with quadruple therapy. However, there are various studies exploring the efficacy of IVIG in preventing thrombotic events in patients with APS. They revealed that patients who received IVIG in addition to conventional treatment had less thrombosis than the group only on classic therapy [16, 17]. In the aforementioned case, we initially used anticoagulation, glucocorticosteroids, therapeutic plasma exchanges and because of the lack of renal function recovery we added the intravenous immunoglobulins in a standard dose. Under this kind of treatment, a definite improvement in the renal function was observed and dialysis could be stopped.

The treatment enumerated earlier does not influence the production of autoantibodies. B cells are not only responsible for their generation after transforming into plasmatic cells, but can also function as antigen presenting cells, differentiate into B cells effectors, regulate helper T cells or can also release pro-inflammatory cytokines [18]. The usage of biologic agents depleting B cells in the CAPS therapy seems to have a strong theoretical foundation. Rituximab (RTX) is the monoclonal antibody directed against the B cell surface marker CD 20 found on pre-B cells maturing to memory B cells. RTX treatment causes a profound depletion of the B cells subset. RTX is the most studied biologic agent in CAPS therapy. “CAPS registry” reports that of 20 patients treated with RTX, 16 (80%) of them survived. RTX was used as a first line therapy in 40% of patients and 60% of them received the drug as a second line therapy because of a poor response to conventional treatment or relapses [9]. The most commonly used dosage was 2 g in two divided doses. The factors identified as being associated with a relapse of the disease are: concomitance of systemic lupus erythematosus, age above 36 years old; pulmonary and renal involvement; the presence of lupus anticoagulant; microangiopathic hemolitic anemia (MHA) [19]. In the presented case we decided to use RTX as a treatment preventing a relapse. The renal involvement with the necessity of renal replacement therapy, the reappearance of lupus anticoagulant and the presence of microangiopathic hemolitic anemia (MHA) were the risk factors presented by our patient which could befall a relapse. Taking into account the life-threatening course of the first episode of CAPS, preventing the relapse was extremely important. The risk of CAPS relapse outweighed the risk of additional immunosuppression. As with most patients with CAPS, our patient also received 2 g of RTX but in three separate doses. The treatment was effective in terms of clinical signs and immunity profile. Over the course of 1 year after the last dose of RTX, no new thrombotic event was observed and the renal function ameliorated. Under the treatment lupus anticoagulant also became negative, however, 1 year after the last dose of RTX it marginally increased to slightly positive. In the pilot study exploring the RTX effects in patients with non-criteria manifestation APS, RTX therapy did not significantly influence the aPL profile. However, it was effective in the treatment of some clinical manifestations of APS [20].

## Conclusion

CAPS is a life-threatening condition and prompt, complex treatment is necessary. The quadruple therapy including drugs acting in a different manner: anticoagulation, glucocorticoids, therapeutic plasma exchanges and intravenous immunoglobulins can capture this thrombotic and inflammatory storm. Rituximab as an immunosuppressive agent with a long-lasting action, can enhance the effect of this therapy and prevent a relapse. Further studies concerning the longitudinal influence of RTX on CAPS survivors are needed.

## Abbreviations

AC: Anticoagulation
ANA: Antinuclear antibodies
aPL: Antiphospholipid antibodies
APS: Antiphospholipid syndrome
APTT: Activated partial thromboplastin time
ASA: Acetylosalicylic acid
aβ2GPI: Anti β2-glicoprotein-I antibodies
CAPS: Catastrophic antiphospholipid syndrome
CT: Computer tomography
GCS: Glucocorticoids
IVIG: Immunoglobulin
LA: Lupus anticoagulant
MHA: Microangiopathic hemolitic anemia
NF: Nuclear factor
RTX: Rituximab
SIRS: Systemic inflammatory response syndrome
SLE: Systemic lupus erythematosus
TMA: Thrombotic microangiopathy
TPE: Therapeutic plasma exchanges
WBC: Leucocytosis

## References

[1] Nayer A, Ortega LM (2014). Catastrophic antiphospholipid syndrome: a clinical review. J Nephropathol.

[2] Espinosa G, Bucciarelli S, Cervera R, Gómez-Puerta JA, Font J (2006). Laboratory studies on pathophysiology of the catastrophic antiphospholipid syndrome. Autoimmun Rev.

[3] Rosário C, Zandman-Goddard G, Meyron-Holtz EG, D'Cruz DP, Shoenfeld Y (2013). The hyperferritinemic syndrome: macrophage activation syndrome, Still's disease, septic shock and catastrophic antiphospholipid syndrome. BMC Med.

[4] Asherson RA, Cervera R, de Groot PG, Erkan D, Boffa MC, Piette JC (2003). Catastrophic antiphospholipid syndrome: international consensus statement on classification criteria and treatment guidelines. Lupus.

[5] Asherson RA, Espinosa G, Cervera R, Font J, Reverter JC (2002). Catastrophic antiphospholipid syndrome: proposed guidelines for diagnosis and treatment. J Clin Rheumatol.

[6] Cervera R, Font J, Gómez-Puerta JA, Espinosa G, Cucho M, Bucciarelli S, Ramos-Casals M, Ingelmo M, Piette JC, Shoenfeld Y, Asherson RA (2005). Catastrophic antiphospholipid syndrome registry project group. Validation of the preliminary criteria for the classification of catastrophic antiphospholipid syndrome. Ann Rheum Dis.

[7] Rodríguez-Pintó I, Moitinho M, Santacreu I, Shoenfeld Y, Erkan D, Espinosa G, Cervera R (2016). CAPS registry project group (European forum on antiphospholipid antibodies). Catastrophic antiphospholipid syndrome (CAPS): descriptive analysis of 500 patients from the international CAPS registry. Autoimmun Rev.

[8] Kazzaz NM, McCune WJ, Knight JS (2016). Treatment of catastrophic antiphospholipid syndrome. Curr Opin Rheumatol.

[9] Cervera R, Rodríguez-Pintó I, Colafrancesco S, Conti F, Valesini G, Rosário C, Agmon-Levin N, Shoenfeld Y, Ferrão C, Faria R, Vasconcelos C, Signorelli F, Espinosa G (2014). 14th international congress on antiphospholipid antibodies task force report on catastrophic antiphospholipid syndrome. Autoimmun Rev.

[10] Berman H, Rodríguez-Pintó I, Cervera R, Morel N, Costedoat-Chalumeau N, Erkan D, Shoenfeld Y, Espinosa G (2013). Catastrophic antiphospholipid syndrome (CAPS) registry project group (European forum on antiphospholipid antibodies). Rituximab use in the catastrophic antiphospholipid syndrome: descriptive analysis of the CAPS registry patients receiving rituximab. Autoimmun Rev.

[11] Pierangeli SS, Girardi G, Vega-Ostertag M, Liu X, Espinola RG, Salmon J (2005). Requirement of activation of complement C3 and C5 for antiphospholipid antibody-mediated thrombophilia. Arthritis Rheum.

[12] Kronbichler A, Frank R, Kirschfink M, Szilágyi Á, Csuka D, Prohászka Z, Schratzberger P, Lhotta K, Mayer G (2014). Efficacy of eculizumab in a patient with immunoadsorption-dependent catastrophic antiphospholipid syndrome: a case report. Medicine (Baltimore).

[13] Oku K, Amengual O, Atsumi T (2012). Pathophysiology of thrombosis and pregnancy morbidity in the antiphospholipid syndrome. Eur J Clin Investig.

[14] Girardi G, Redecha P, Salmon JE (2004). Heparin prevents antiphospholipid antibody-induced fetal loss by inhibiting complement activation. Nat Med.

[15] Auphan N, DiDonato JA, Rosette C, Helmberg A, Karin M (1995). Immunosuppression by glucocorticoids: inhibition of NF-kappa B activity through induction of I kappa B synthesis. Science.

[16] Tenti S, Guidelli GM, Bellisai F, Galeazzi M, Fioravanti A (2013). Long-term treatment of antiphospholipid syndrome with intravenous immunoglobulin in addition to conventional therapy. Clin Exp Rheumatol.

[17] Sciascia S, Giachino O, Roccatello D (2012). Prevention of thrombosis relapse in antiphospholipid syndrome patients refractory to conventional therapy using intravenous immunoglobulin. Clin Exp Rheumatol.

[18] Khattri S, Zandman-Goddard G, Peeva E (2012). B-cell directed therapies in antiphospholipid antibody syndrome-new directions based on murine and human data. Autoimmun Rev.

[19] Bucciarelli S, Erkan D, Espinosa G, Cervera R (2009). Catastrophic antiphospholipid syndrome: treatment, prognosis, and the risk of relapse. Clin Rev Allergy Immunol.

[20] Erkan D, Vega J, Ramón G, Kozora E, Lockshin MD (2013). A pilot open-label phase II trial of rituximab for non-criteria manifestations of antiphospholipid syndrome. Arthritis Rheum.

